# 

**Published:** 1890-05-24

**Authors:** 


### NEW OFFICES IN THE STRAND.

The Hospital *is now published at* **140, Strand, W.C.** *It has been found desirable to establish* The Hospital *in this central and convenient locality, owing to the rapid extension of the business of this paper of late.*

### NOTICE TO CORRESPONDENTS.

All MS., Letters, Books for Review, and other matters intended for the Editor should be addressed The Editor, The Lodge, Porchester Square, London, W.

The Editor cannot undertake to return rejected M.S., even when accompanied by stamped directed envelope.

### Notice to Advertisers and Subscribers.

All Advertisements, Orders for Copies of The Hospital, and Business Communications should be addressed to The Publisher, *not to the Editor*, The Hospital, 140, Strand, W.C.

### Bound Volumes of "The Hospital."

Vol. VI., containing full page portraits of T.R.H. the Prince and Princess of Wales, and Miss Florence Nightingale, is obtainable at 4s., post free.

Orders will be received for Vol. VII., 4s. post free. Cases for binding may be had of the Publisher, at 1s. 6d. each.

### To Residents, Secretaries, and Matrons.

The Editor will be glad to have the Regulations and each Report as issued by Asylums, Hospitals, Convalescent and Nursing Institutions, as well as those of other Charities, and an early copy in duplicate of all Appeals and Festival Notices, etc., addressed to *The Editor, The Lodge, Porchester Square, London, W.* Any superintendent, secretary, matron, or other chief official who regularly sends such information may be registered, on application to the Publisher, to receive free of cost a cover for binding The Hospital in half-yearly volumes.

### Scraps and Gleanings.

The Lord Mayor took the chair on Wednesday at a meeting for furthering smoke abatement.

Inverness Infirmary issues a good report. They hope to have a convalescent branch to the institution soon.

Princess Christian will preside at a meeting of the Cyprus Society, to be held on June 2nd, at 24, Park Lane.

The Marchioness of Waterford has lent her house in aid of a meeting for the East-end Mothers' Home, to be held on June 4th.

Glasgow Ear Hospital treated 900 patients last year—an increase of 80 on the previous year. Dr. Thomas Barr is the honorary surgeon.

David Nathan, the oldest survivor of the battle of Leipsic, who died at the age of 101, was buried, last week, at Hamburg, with military honours.

A large number of cases of typhoid exists in Cairo, amounting to a veritable epidemic, caused, it is said, by the impurity of the water. The hospital at Abassieh is nearly full.

The recently constituted Working Men's Hospital Committee at Grimsby does not seem to have got into working order yet, for the attendance at the late meeting was very poor.

Mr. George Griffith has been censured by the Pembrokeshire Infirmary Committee for sending an incurable patient to the infirmary. Would it not be wise for the committee to find a less humane fault before censuring their senior surgeon.

Mr. Thomas Jennings, "the fat man of Tasmania," died on April 1, at the age of sixty-six. He was 5 ft. 10 in. in height, and weighed 33 stone. His chest measured 68 inches, waist 82, and calf 21½. He was a native of Stream Head, Alberton, Yorkshire.

The return of metropolitan pauperism for the fifth week of April states that the total number of paupers was 92,179. The number in the corresponding period of 1889 was 94,628: of 1888, 101,694; and of 1887, 95,654. The number of vagrants relieved during the same period was 857, including 648 men, 185 women, and 24 children.

Professor Schweninger, the chief physician of Prince von Bismarck, is teaching two Turkish physicians, Fahry Bey and Berim Bey, his famous cure for corpulency. These Oriental pupils will treat the Sultan on their return to Constantinople. His Ottoman Majesty desired Professor Schweninger to attend him personally, but the latter is too busy to leave Germany.

